# Anti-inflammatory, Antipruritic and Mast Cell Stabilizing Activity of *Aristolochia Indica*

**Published:** 2011

**Authors:** Jessy Elizabeth Mathew, Srinivasan Keloth Kaitheri, Seekarajapuram DinakaranVachala, Magi Jose

**Affiliations:** 1 *Department of Pharmaceutical Chemistry, MCOPS, Manipal University, Karnataka, India*; 2 *Department of Oral Pathology, Yenepoya University, Mangalore, Karnataka, India*

**Keywords:** Anti-inflammatory, Anti-antipruritic, Aristolochia indica, Mast cell stabilizing activity

## Abstract

**Objective(s):**

*Aristolochia indica* has been widely used in the traditional medicine for the treatment of a variety of diseases. In the present study different extracts of roots of* A. indica *were evaluated for their anti-inflammatory, antipruritic and mast cell stabilizing activity.

**Materials and Methods:**

Anti-inflammatory activity was performed by compound 48/80 induced rat paw edema model and antipruritic activity by examining the incidence of scratching behavior. Mast cell stabilizing activity was performed by compound 48/80 and sheep serum induced mast cell degranulation methods.

**Results:**

The ethanol extract (300 mg/kg) and petroleum ether extract (100 mg/kg) were found to inhibit mast cell degranulation significantly equivalent to that of standard drug ketotifen (69%) by compound 48/80 model. In sheep serum model the ethanol extracts (150 and 300 mg/kg) and petroleum ether extract (100 mg/kg) showed good mast cell stabilizing activity (66-67%). Ethanol extract at 150 mg/kg showed 70% reduction of rat paw oedema and also significantly reduced the scratching response.

**Conclusion:**

Results suggest *A. indica *has good mast cell stabilizing, anti-inflammatory and antipruritic activity.

## Introduction

*Aristolochia *Linn (Aristolochiaceae) is a large genus of herbs or twinning plants comprising about 300 species found in the tropical and temperate regions of the world (Brazil, Texas, Europe etc.). Eight species are known to occur in of which *Aristolochia braccteolata*, *A. indica*, and *A. tagala* are of medicinal importance. They generally contain alkaloids and are reported to be useful in the treatment of snakebites (1). * A. indica* is a perennial- climbing shrub commonly found in . The main constituents are aristolochine and aristolochic acid (2). Methyl ester of aristolochic acid was isolated from the roots of *A. indica* and is used as an abortifacient (3). Aristoloside was reported to inhibit carcinogenesis (4). Aristolochic acid was reported to possess various biological activities such as antibacterial, anti- inflammatory, antiadenocarcinoma, antineoplastic (5), antitumor activities (6) and antiviral (7). *A. indica* was used by the tribes like Siddis (8) and Gowlis (9) for the treatment of skin diseases. A survey of literature revealed that no systematic approach has been made to study the usefulness of this plant for various skin aliments including, antiallergic, anti-inflammatory and antipruritic activities. Thus, the present study was undertaken to assess the effect of this indigenous herb on different parameters related to antiallergic activity in rats and also to study the anti-inflammatory and antipruritic activity. 

## Materials and Methods

***Plant material***

Fresh roots of *A. indica* were collected from in and around Udupi, , , in September 2007 and were authenticated by Prof. Gopalakrishna Bhat, Department of Botany, , Udupi. A voucher specimen No.PP-605 has been deposited at the Department of Pharmaceutical Chemistry, , Manipal.

***Chemicals***

Compound 48/80, toluidine blue, RPMI-1640 were purchased from sigma chemicals Ltd, . Ketotifen fumarate was obtained from Ltd., Mumbai. Sheep serum was collected from the local slaughter house under sterile conditions. Triple antigen was procured from Biological E Ltd.

***Preparation of ethanol extract***

The shade dried powdered stem bark (2 kg) was exhaustively extracted in a Soxhlet apparatus with 95% ethanol. The total ethanol extract was concentrated in vacuo to a syrupy consistency (yield 340 g). The crude ethanol extract was fractionated with solvents of different polarity. The semisolid extract was uniformly suspended in distilled water (500 ml) and exhaustively extracted with petroleum ether, diethyl ether, ethyl acetate, ethyl methyl ketone, n-butanol and finally with water. All the fractions were washed with distilled water and dried over anhydydrous sodiumsulphate and freed off from solvent by distillation under reduced pressure. The aqueous remnant was concentrated by evaporation on water bath slowly to dryness. 

***Animals***

Healthy adult male albino rats (150-200 g) of Wistar strain were used for the study. The animals were maintained under standard environmental conditions and had free access to standard diet (Hindustan Lever Ltd.) and water *ad libitum*. Study was conducted after obtaining institutional animal ethical committee clearance No.IAEC/KMC/10/2001.

***Acute toxicity studies***

The rats in groups of 6 each were fed with ethanol extract of *A. indica* suspended in acacia gum (2% w/v) at increasing dose levels of 0.5, 1, 2 and 3 g/kg body weight. The animals were observed continuously for 2 hr for gross behavioral changes and then intermittently once every 2 hr and finally at the end of 24 hr and 72 hr (10).

***Compound 48/80 induced rat paw edema***

Inflammation was induced in rats by the injection of compound 48/80 (p-methoxy-N-methyl-phenylamine; 0.5 ml of 10 µg/ml in normal saline) into the sub-plantar tissue of the right hind paw. The linear paw circumference was measured at half an hour intervals for 3 hr. The animals were dosed once daily for 7 days prior to study; the extract (150 mg/kg) and ketotifen fumarate (1 mg/kg) were administered orally, 1 hr before induction of inflammation. Control animals received an equal volume of 2% w/v acacia (11).

***Assay of antipruritic activity***

The antipruritic activity was evaluated by examining the incidence of scratching. Scratching behaviour was induced by subcutaneous injection of 0.1% solution of compound 48/80 in saline at 100 µl/site into the base of the neck on the back side of the rat. Scratching on the injected site by the hind paws were counted for 30 minutes disregarding those at other sites such as ears. Test compound such as ethanol extract of A. indica (150 mg/kg) was given orally 1 hr before the injection of the compound 48/80. As a control, rats were administered 2% gum acacia (2 ml) orally. Chlorpheniramine maleate was used as reference standard (12).

***Compound 48/80 induced systemic anaphylaxis***

Compound 48/80 induced systemic anaphylaxis was carried out according to the previous method. Briefly, rats were given an intraperitoneal injection of 8 mg/kg body weight of compound 48/80. Test doses were given orally at doses of 50 mg to 1000 mg, 1 hr before injection of compound 48/80. Mortality was monitored for 1 hr after the induction of anaphylactic shock (13).

***Mast cell stabilizing activity Compound 48/80 induced allergy test***

Compound 48/80 is a powerful histamine releasing agent from the mast cells. Healthy adult albino rats of either sex of Wistar strain weighing between 150-200 g were selected for the study. Test extracts were given orally to overnight fasted animals. The animals were divided randomly into seven group (n= 6) and were given different doses of the extracts of *A.* *indica* by oral route (14-15).

***Sheep serum induced allergy model***

Sheep serum induced allergy test was performed on healthy adult albino rats (16). Allergy was induced in overnight fasted animals by a single subcutaneous injection of 0.5 ml of sheep serum along with 0.5 ml of triple antigen. The animals were divided randomly into seven group (n= 6) and were given different doses of the extracts of *A.* *indica* by oral route.

The sensitized rats were divided into seven groups of six animals.

Group I: control, received only vehicle (2% solution of gum acacia, orally 2 ml / kg p.o.).

Group II: Treated with ketotifen fumarate (1mg/kg p.o.).

Group III / IV: Treated with ethanol extract of *A. indica* (150 and 300 mg/kg p.o.).

Group V: Treated with petroleum ether extract of *A. indica* (100 mg/kg p.o.)

Group VI: Treated with ethyl acetate extract of *A. indica* (100 mg/kg p.o.)

Group VII: Treated with aqueous extract of *A. indica* (100 mg/kg p.o.)

## Results

Acute toxicity studies showed the nontoxic nature of the ethanol extract of *A. indica*. There was no lethality or any toxic reactions found at any of the doses selected until the end of the study period. 

Different extracts of *A. indica* were studied for their anti-inflammatory, antipruritic and mast cell stabilizing activity. The ethanol extract of *A. indica* at 150 mg/kg dose showed significant reduction of rat paw edema (70%) (Table 1). Antipruritic activity was studied on compound 48/80 induced scratched behavior model. Subcutaneous injection of compound 48/80 elicited a significant scratching response in mice. The average scratching frequency in the 10 min after the injection of compound 48/80 was 76.66 ± 2.70. *A. indica* ethanol extract at dose of 150 mg/kg significantly reduced the scratching response which was comparable to that of chlorpheneramine maleate (Table 2). Systemic anaphylactic shock was induced by compound 48/80 in rats.

**Table 1.** Effect of ethanol extract of the root bark of *Aristolochia indica* on anti-inflammatory activity induced by compound 48/80.

Treatment	Dose	% Inhibition			
		30 min	60 min	120 min	180 min
Ketotifen fumarate	1mg/kg	53.83±2.02	60.50±1.32	70.16±1.44	71.83±2.42
Ethanol Extract	150mg/kg	47.83±1.51	57.50±2.48	68.83±1.21	70.50±1.71

**Table 2.** Effect of ethanol extract of the root bark of *Aristolochia indica* on antipruritic activity induced by compound 48/80.

Treatment	Dose	No. of animals	Incidence of scratching
Control	2% gum acacia	6	76.66±2.70
Chlorpheniramine maleate	0.325 mg/kg	6	35.50±1.32*
Ethanol extract	150 mg/kg	6	36.33±1.15*

* **P** ***<*** ***0.05 vs control (ANOVA post hoc Scheffe’s test).***

An intraperitoneal injection of compound 48/80 (8 mg/kg) resulted in 100% fatal shock. *A. indica* pretreatment at doses ranging from 50 to 500 mg/kg 1 hr before the injection of compound 48/80, dose dependently reduced the mortality rate (Table 3). Effect of various extracts of *A. indica* on mast cell stabilization was studied by compound 48/80 and sheep serum induced allergy models. Ethanol extract 300 mg and petroleum ether extract 100 mg/kg 

showed significant mast cell stabilizing activity (69%) on compound 48/80 induced allergy model. The activity was comparable to that of standard drug ketotifen. The results were depicted in Table 4. Ethanol extract at the doses of 150 and 300 mg/kg and petroleum ether at the dose of 100 mg/kg showed significant protection against mast cell degranulation induced by sheep serum (66 to 67%) (Table 5). 

**Table 3.** Effect of *Aristolochia indica* root bark ethanol extract on compound 48/80 induced systemic anaphylaxis.

Treatment	Compound 48/80 (8 mg/kg)	Mortality (%)
Control (2 ml saline)	+	100
Ethanol extract (50 mg/kg)	+	100
Ethanol extract (100 mg/kg)	+	80
Ethanol extract (150 mg/kg)	+	50
Ethanol extract (300 mg/kg)	+	20
Ethanol extract (500 mg/kg)	+	-
Ketotifen fumarate (1 mg/kg)	+	-

**Table 4.** Effects of different extracts of the root bark of *Aristolochia indica* on mast cell degranulation induced by the antigen compound 48/80.

Group	Treatment	Dose	No.of animals	Mast cells%	
				Intact	Disrupted
1	Control	2% solution	6	22.00±2.04	72.91±0.49
2	Ketotifen fumarate	1 mg/kg	6	69.50±2.85*	30.42±1.84
3	Ethanol extract	150 mg/kg	6	66.00±2.56*	34.14±2.72
4	Ethanol extract	300 mg/kg	6	69.16±1.21*	31.30±2.33
5	Petroleum ether extract	100 mg/kg	6	69.50±1.32*	30.31±3.8
6	Ethyl acetate extract	100 mg/kg	6	40.16±3.58*	60.38±1.88
7	Water extract	150 mg/kg	6	40.50±1.32*	59.91±0.69

*P< 0.05 vs control (ANOVA post hoc Scheffe’s test).

**Table 5.** Effect of different extracts of the root bark of *Aristolochia indica* on mast cell degranulation induced by an antigen sheep serum.

Group	**Treatment**	Dose	No.of animals	Mast Cells %	
				Intact	Disrupted
1	Control	2% solution	6	22.16±1.29	77.78±1.29
2	Ketotifen fumarate	1 mg/kg	6	75.33±2.99*	24.60±3.37
3	Ethanol extract-I	150 mg/kg	6	66.33±1.46*	33.13±3.64
4	Ethanol extract-II	300 mg/kg	6	67.33±2.08*	32.11±2.86
5	Petroleum ether extract	100 mg/kg	6	66.00±2.06*	34.86±3.41
6	Ethyl acetate extract	100 mg/kg	6	23.83±2.58	76.61±1.09
7	Water extract	150 mg/kg	6	38.00±1.73	62.54±3.01

*P< 0.05 vs control (ANOVA post hoc Scheffe’s test).

## Discussion

In acute toxicity studies all extracts were found to be safe in the doses used and there was no mortality up to a dose of 3000 mg/kg, p.o. Effects of various extracts of entire plant were studied on sheep serum induced mast cell degranulation and compound 48/80 induced mast cell degranulation. A mast cell is a resident cell of several types of tissues and contains many granules rich in histamine. Although best known for their role in allergy and anaphylaxis, mast cells play an important protective role as well, being intimately involved in wound healing, anti-inflammatory activity and defense against pathogens (17). 

An antiallergic activity of *A. indica* might be contributed by inflammatory mediator inhibitory pathway. *A. indica* significantly inhibited the compound 48/80 induced scratching, cutaneous inflammation and anaphylaxis. Anaphylaxis is a severe and systemic allergic reaction caused by systemic release of histamine and other inflammatory chemical mediators. One of the newer methods of anaphylaxis treatment involves use of immunotherapeutic agent by decreasing production of IgE. In the present study, ethanolic extracts of *A. indica* showed dose dependent protection against compound 48/80 induced anaphylaxis up to 100%. 

Antipruritic activity was evaluated by observing the incidence of scratching behavior by subcutaneous administration of compound 48/80. Compound 48/80 alone administered had administration of compound 48/86 alone significantly increased the number of scratching and thought to be associated with release of histamine from mast cell degranulation. However, the ethanolic extract of *A. indica* was useful in the treatment of most of the allergic diseases (12).

Effect of various extracts of *A. indica* on mast cell stabilization was studied by compound 48/80 and sheep serum induced allergy models. Ethanol extract 300 mg and petroleum ether extract 100 mg/kg showed significant mast cell stabilizing activity (69%) on compound 48/80 induced allergy model. The activity was comparable to that of standard drug ketotifen. The results were depicted in Table 4. Ethanol extract at the doses of 150 and 300 mg/kg and petroleum ether at the dose of 100 mg/kg showed significant protection against mast cell degranulation induced by sheep serum (66 to 67%) (Table 5).

In case of mast cell stabilizing activity the drug probably acted by stabilization of mast cell membrane. Possibly the nitric oxide synthase inhibitory activity was also responsible for the antiallergic activity. Stimulation of mast cells with compound 48/80 or antiserum initiates the activation of signal transduction pathway, which leads to histamine release. Some recent studies showed that compound 48/80 and other polybasic compounds are able to activate G proteins (18). The compound 48/80 increased the permeability of the lipid bilayer membrane by causing a perturbation of the membrane. These results indicated that the membrane permeability increase may be an essential trigger for the release of the mediators from the mast cells. More recently studies have suggested the importance of chloride channels that provide the driving force for calcium influx during mast cell activation. 

## Conclusion

The promising antipruritic, anti-inflammatory activities recorded for the ethanolic extract has eresults provide ample justification for the claims made in the indigenous system of medicine for *A. indica*. 
